# Children in Vegetative State and Minimally Conscious State: Patients' Condition and Caregivers' Burden

**DOI:** 10.1100/2012/232149

**Published:** 2012-02-01

**Authors:** A. M. Giovannetti, M. Pagani, D. Sattin, V. Covelli, A. Raggi, S. Strazzer, E. Castelli, A. Trabacca, A. Martinuzzi, M. Leonardi

**Affiliations:** ^1^Neurology, Public Health and Disability Unit, Scientific Directorate, Neurological Institute Carlo Besta IRCCS Foundation, 20133 Milan, Italy; ^2^IRCCS “E. Medea”, Association “La Nostra Famiglia”, Centre of Bosisio Parini, Via Don Luigi Monza 20, 23842 Bosisio Parini, Italy; ^3^IRCCS Ospedale Pediatrico Bambino Gesù, Via Torre di Palidoro, 00050 Passoscuro, Fiumicino, Rome, Italy; ^4^IRCCS “E. Medea”, Association “La Nostra Famiglia”, Centre of Ostuni, Via dei Colli 5-7, 72017 Ostuni, Italy; ^5^IRCCS “E. Medea”, Association “La Nostra Famiglia”, Centre of Conegliano-Pieve di Soligo, Via Monte Grappa 96, 31053 Treviso, Italy

## Abstract

Caring for children in vegetative state (VS) or minimally conscious state (MCS) challenges parents and impacts on their well-being. This study aims to evaluate caregivers' health condition, coping, anxiety and depression levels, and how these issues relate to children's disability. 35 children with VS and MCS were administered the disability rating scale (DRS) and 35 caregivers completed the Coping Orientations to Problem Experiences, Short Form-12, Beck Depression Inventory, and the Spielberger State-Trait Anxiety Inventory-Y. Children were mainly males (68.6%), hosted at domicile (77.1%), and diagnosed with VS (60%), with anoxic aetiology (45.7%). Caregivers were mainly mothers (85.7%), married (82.9%), and housewives (51.4%); 60% declared financial difficulties, and 82.9% provided full-time assistance. 57.2% reported depressive symptoms, poor mental health, and high level of state and trait anxiety. “Problem-oriented” (*P* < 0.001) and “emotional-oriented” (*P* < 0.001), were more adopted than “potentially dysfunctional” ones. DRS scores (mean = 22.0; SD = 1.9) did not significantly correlate to any psychological measure. Rehabilitative programs for children with SV and SMC should also provide interventions on surrounding systems: improving the network of psychological support and social assistance may decrease the burden of caregivers and, in turn, improve caring abilities and children quality of life.

## 1. Introduction

Vegetative state (VS) and minimally conscious state (MCS) are possible outcomes after an acquired severe brain injury (ASBI), encompassing traumatic and nontraumatic brain injuries, clinically included in the group of disorders of consciousness (DOCs) [1]. Even though patients in VS are in a condition of wakefulness, they are unable to show any residual behavioural evidence of awareness of themselves and of the environment [2]. MCS is instead defined as a state in which daily or clinical observation can bring to notice some residual finalistic behavioural signs of awareness, including following basic orders, staring and eye tracking, or using objects functionally [3].

With regards to recent advance in emergency medicine and intensive care techniques, the number of patients surviving ASBI has been gradually increasing. Incidence and prevalence of these conditions range from 0.5 to 4/100.000 and from 0.6 to 10/100.000 inhabitants, respectively [4]. Even though precise epidemiological data upon children with VS or MCS are not available, a study about life expectancy of VS and MCS children emphasised that these conditions are long standing and showed that 63% to 81% of the patients survive at least 8 years. It was also demonstrated that acquired brain injury in childhood and greater motor responses are better predictor of survival than the severity of level of consciousness [5].

Caring for a relative with a chronic disease [6–9] and in particular providing care for a child with severe disability are recognized as a risk factor for decreased physical and psychological health, with effects in terms of well-being and burden of caregivers [10]. Burden is a complex construct encompassing physical, psychosocial, and financial dimensions which, in the frame of the present paper, are considered related to care-giving activities. In a public health perspective, caregivers necessitate emotional and financial support from public sectors [11], hence, burden impacts on the society and entails practical consequences on health management and welfare policy. Children with VS and MCS have different clinical profiles; however, they both require constant and prolonged assistance that leads families to essential life changes. As reported by Raina et al. [10], caregivers show a considerable variation in the adaptation to their role's demands, depending both on child health state and parents' intrapsychic factors. Research conducted on patients with cancer, heart failure, and chronic obstructive pulmonary disease suggested that burden can be considered as a consequence of caregiver's ability to adapt to the care-giving role, rather than being associated to real patient's needs for assistance [12].

Taking care of a child is part of the natural parental role. However, when a child has severe functional impairments and limitations that lead to an almost complete dependence from environment, caregiving is likely to assume different significances. Providing care for a child with chronic disorders and severe disability is an additional role that necessitates a redefinition of priorities and use of time and financial resources and can consequently contribute to increased level of perceived burden [13]. Providing care for people with long-term diseases becomes a dynamic process, where several goals must be accomplished through mechanisms of adaptation and change of responsibilities over time, including acquisition of care-giving role, performance of daily tasks, and eventual end of the role connected to child's death [14–16].

As reported in the literature, caring for DOC patients is considered as an emotional paradox difficult to elaborate and cope with, which makes caregivers feel imprisoned in a sense of guilt, regrets, and memories, determining inability to react to this stressful event [17]. Despite the paucity of studies assessing burden of caregivers of patients with VS or MCS, and the lack of studies on these conditions in childhood, the role played by psychological and social components, such as anxiety, depression, and coping strategies, has been emphasized [18–20]. Previous studies on VS patients' caregivers reported severe and stable levels of emotional distress and anxiety or depressive symptoms over time as relevant component of burden [19–21]. Coping strategies were also identified as significant to modify the relationship between stressful life events and personal functioning [22]; in particular, it has been reported that emotion-focused strategies may prevent caregivers of patients with severe chronic conditions from developing anxiety levels, more effectively than problem-focused strategies [23].

In sum, the paucity of data on the complex condition of children with DOCs, and the absence of studies specifically targeted on the effect of having a child with a severe chronic disability on caregivers' health and emotional status, highlighted the need to better understand caregivers' burden in connection with children's conditions. Therefore, the aim of this paper is to describe children impairments, evaluate caregivers' health state, their coping strategies, anxiety and depression levels, and their relationships to children's level of disability.

## 2. Materials and Methods

This observational multicentre cross-sectional study was conducted as a national survey, and it was supported by a grant of Italian Ministry of Health. The study enrolled children in VS and MCS, aged under 18, and hosted in post-acute rehabilitation heath care facilities or at domicile and their caregivers. Children were enrolled at home if they were regularly followedup by staff members of an institution devoted to care and rehabilitation of DOCs. Main caregivers, that is, the person mainly involved in terms of time with the patient for informal caring and felt responsible for the patient, were enrolled as participants.

The protocol for children with disorders of consciousness and for their caregivers was presented by trained psychologists and medical doctors of each institution and required about 100 minutes to be completed. It was composed of a clinical scale to assess children level of disability and a self-reported battery of questionnaires to evaluate caregivers' burden. A brief introduction on instrumentation, modalities, and aims was also included. Participation was on a voluntary basis, and short breaks were allowed to attenuate fatigue effects. Informed consent was provided, and confidentiality was preserved. The study was approved by the local ethics committee.

### 2.1. Measures

The disability rating scale (DRS) used for children is an 8-item reliable and valid clinical scale to assess impairment and disability of patients with acquired brain injuries. DRS is completed by trained health professionals on the basis of objective evaluation and is intended to accurately measure general functioning in different stages of the course of recovery. It assesses eye opening, communication ability, motor response, feeding, toileting, and level of functioning and employability. Scores range from 0 to 29, and higher values indicate higher disability [24].

Caregivers were administered the following self-report tools: a sociodemographic questionnaire, the Coping Orientations to Problem Experiences (COPE) [25], Short Form-12 (SF-12) [26, 27], the Beck Depression Inventory (BDI- II) [28], and the Spielberger State Trait Anxiety Inventory-Y (STAI-Y) [29].

COPE is a 60-item scale used to evaluate 15 strategies adopted to cope with stressful situations. They are grouped into 3 main factors, namely, “problem-oriented strategies” (20 to 80), concerning active coping, planning, suppression of competing activity, restraint coping, and seeking social support for instrumental reason, “strategies of emotional expression” (24 to 96), concerning seeking social support for emotional reason, focusing on venting of emotions, positive reinterpretation, acceptance, turning to religion, and humour, “potentially dysfunctional strategies” (16 to 64), concerning denial, behavioural, mental, and substance disengagement. Higher scores suggest higher frequency of usage of the strategy.

SF-12 is a self-report questionnaire that was used to describe caregiver general health conditions. It investigates a wide set of domains, including vitality, physical functioning, bodily pain, general health perception, mental health, and physical, emotional, and social role functioning. These characteristics are clustered in 2 factors, namely, “physical component summary” (PCS, ranging from 11 to 70), describing level of limitations in self-care, physical, social, and caregiver role activities, bodily pain, tiredness, disabilities, well-being and energy level, and “mental component summary” (MCS, ranging from 7 to 72), describing social and role disability due to emotional problems and psychological distress. Higher scores indicate better-perceived health condition.

BDI-II is a 21-item self-report inventory that was used to evaluate the presence and severity of depressive symptoms. It investigates manifestations of depression, including pessimism, guilt, self-criticism and self-esteem, loss of interest and energy, changes in appetite and sleep, agitation, and crying. BDI-II provides two subscores, namely, a cognitive and a somatic-affective score, as well as a global index ranging from 0 to 63, with higher scores suggesting more severe symptoms. According to percentile ranges, severity of depressive symptoms is defined as minimal (lower than 85 percentiles), mild (between 85 and 90), moderate (between 91 and 95) and severe (higher than 95).

STAI-Y is an inventory that measures feelings of apprehension, tension, nervousness, and worry. 2 main factors are independently calculated to differentiate between the temporary condition of anxiety or “state anxiety”, and the more general and long-standing property of anxiety or “trait anxiety.” STAI-Y is composed of 40 items ranging from 1 to 4, and both “state anxiety” and “trait anxiety” scores range from 20 to 80. Higher scores indicate higher level of anxiety.

### 2.2. Statistical Analysis

Descriptive statistics were performed to illustrate the distribution of sociodemographic, psychological and clinical variables. As each factor of COPE is composed of a different number of items, a linear transformation (new COPE factor score = raw COPE factor score/number of items of the factor) was executed to allow comparison between factor scores. Comparisons between factor scores among the entire sample were performed using within-subjects *t*-tests, with the application of Bonferroni's correction for multiple comparisons. One-sample *t*-tests were calculated to compare STAI-Y (separately for men and women) and SF-12 mean scores to the respective normative sample. The relationships between children clinical assessment and caregivers' psychological evaluation were evaluated using Pearson's product moment coefficient. Significance level *α* = 0.05 was adopted, and all statistical analyses were performed using SPSS v. 11.0.

## 3. Results

A total of 35 children with DOCs and 35 caregivers were enrolled as participants. The majority of the patients were males and living at home, and 60% of the sample were diagnosed with VS and, tetraplegia, dysphagia, and epilepsy were the most common comorbidities reported. Anoxia was the most represented aetiology, and principal rehabilitative interventions consisted in physiotherapy, swallowing and language therapy. Detailed sociodemographic and clinical characteristics are reported in Tables 1 and 2.

The characteristics of caregivers are reported in Table 3. The majority of caregivers were mothers, married, housewife, and with a high school diploma or higher. Over 80% of caregivers provided care for their child 24 hours per day and less than 40% access to psychological support or social assistance. 62.9% of the families reported having a sufficient income, 60% declared financial difficulties, and slightly less than 50% received familiar support. The results of psychological tests are reported in Table 4. Mean BDI-II scores were 14.8 (SD 9.9), somatic-affective component mean score was 10.1 (SD 6.2), and cognitive component mean score was 4.7 (SD 4.8); 57.2% of caregivers exceeded the threshold of 85 percentile and reported at least mild depressive symptoms.

One-sample *t*-test showed that SF-12's PCS was in line with the normative sample (*t* = 0.530, *P* = 0.600), whereas MCS was statistically significantly lower (*t* = −2.119, *P* = 0.041). As far as STAI-Y is concerned, caregivers reported higher level of state anxiety (mean = 45.1, SD = 13.1, *t* = 2.176, *P* = 0.038 for mothers; mean = 48.4, SD = 6.4, *t* = 4.315, *P* = 0.013 for fathers) and trait anxiety (mean = 47.1, SD = 11.0, *t* = 2.892, *P* = 0.007 for mothers; mean = 48.0, SD = 6.6, *t* = 3.932, *P* = 0.015 for fathers). Within-subjects *t*-tests showed that caregivers tended to use potentially dysfunctional strategies less than problem oriented strategies (*t* = −16.934, *P* < 0.001) and strategies of emotional expression (*t* = −21.821, *P* < 0.001).

DRS scores did not significantly correlate to BDI-II (*r* = 0.28, *P* = 0.10), SF-12 (*r* = −0.20, *P* = 0.24 for PCS; *r* = −0.25, *P* = 0.16 for MCS), STAI-Y (*r* = 0.04, *P* = 0.84 for state anxiety; *r* = 0.12, *P* = 0.49 for trait anxiety), and COPE (*r* = 0.15, *P* = 0.40 for problem-oriented strategies; *r* = 0.04, *P* = 0.85 for strategies of emotional expression; *r* = −0.04,  *P* = 0.82 for potentially dysfunctional Strategies) scores.

## 4. Discussion

In line with findings related to adult population with DOCs, this study confirmed the presence of impairments and high levels of disability of VS and MCS children, with high frequency of comorbidities. Our findings also demonstrated the impact of psychological burden of caregivers in terms of mental health status, anxiety, and depressive symptoms, but failed in establishing any correlation between children's level of functioning and family members' burden.

Uncertainty of patient's capability of perceiving the environment and being self-aware as well as poor scientific agreement on actual functioning and prognostic factors makes VS and MCS a paradigm of complexity from a clinical perspective. Multiple co-morbidities detected in this sample, especially tetraplegia, dysphagia, and epilepsy, constitute remarkable aspects that add complexity on the health status. Our findings also showed that these children are mainly diagnosed with VS as a consequence of anoxia, an aetiology that involves wide and diffuse brain damage associated with negative prognostic outcomes and functioning [30].

Bringing up, protecting, and safe-guarding a child represents a natural side of parental role. This role is usually carried out by mothers, but it is interesting to notice that in this sample almost the 15% are fathers. However, when a child has severe functional impairments and limitation that lead to an almost complete dependence from environment, providing care assumes a significance that goes far beyond conventional parental caring [10]. In fact, our findings showed high level of psychological burden of caregivers in terms of mental health status, anxiety, and depressive symptoms.

This sample of patients in VS and MCS necessitates daily assistance as they are not self-sufficient, beyond developmental physiological steps, in basic skills including simple motor response, feeding, toileting, and communication, and parents look after them by providing continuous assistance, including informal and affective support. As our population of children with DOCs mainly lived at home, parents were even more in charge to coordinate all the interventions and activities involving the child—such as rehabilitation, nursing assistance, and generic care activities—than parents of patients hosted in rehabilitation nursing or health care institutions where these duties are generally shared with health care professionals.

Children show severe impairments with tetraplegia, low level of responsiveness, and absence or fluctuating meaningful behaviours, and parents only sporadically receive concrete and tangible feedbacks on their efforts of parental caring. This results in a difficult interpretation of child behaviours and needs beyond those strictly connected to basic daily care, such as nourishment and hygiene. Lack of contact or misinterpretation of child behaviour may cause frustration and contribute to increase the level of burden, in terms of health status, anxiety, and depression. However, based on our data, we can only hypothesize such a relationship, since the results of our study did not provide any indication to defining such a correlation. Further studies should investigate the extent of this “coordination role” in condition of absence of patient's feedback, on caregiver well-being in terms of burden.

Financial conditions are also likely to contribute to reported burden. In fact, the majority of parents complained financial difficulties, declaring only a sufficient income, and resigned from job temporarily or permanently due to patient's health condition. Lack of broader familiar support for caring is a complaint in more than 50% of the cases, and results demonstrated that principal caregivers dedicate the majority of their life to care for their child and they do not feel as being adequately supported.

As argued by Juozapavicius, lack of time for themselves due to care-giving role limitation is one of the main causes of caregiver disorders [31]. In addition, Skaff reported that burden of caregivers with Alzheimer's disease can be strictly related to caregivers' engulfment, indirectly evaluated by two factors: social contact with friends and acquaintances during leisure time and number of roles, such as at work, in the family, and in the community [32]. Considering mental health status, anxiety, and depressive symptoms reported by caregivers' sample, and the general tendency to resign from job, it may be evinced that playing the role of caregiver only affects well-being and overall quality of life.

Even though high-quality perceived physical health was observed, caregivers reported poor perceived mental outcomes, which provides preliminary suggestions to hypothesize that caregiver role significantly affects and has consequences on the mental health. In fact, caregivers showed high levels of both state and trait anxiety compared to the Italian normative sample, and almost a quarter reported pervasive depressive symptomatology. On the other hand, potentially dysfunctional strategies are found to be less frequently used than emotional expression and problem-oriented strategies to cope with this stressful situation, indicating a positive element. In a previous study, Cooper and colleagues [23] demonstrated that emotion-focused coping strategies tend to protect caregivers from developing dysfunctional anxiety, and, therefore, psychological treatments aiming to develop such a strategy may reduce anxiety in caregivers.

Our caregivers' sample did not show any significant differences in the use of emotional expression and problem-oriented strategies, which may be interpreted as negative prognostic factor, that could necessitate a specific intervention, as previously reported for caregivers of patients with other conditions. Findings on caregivers of children with cerebral palsy showed that psychological well-being of caregivers is ascribable to and predicted by the array of manifest behavioural symptoms of children and care-giving demands [10]. Despite the high level of emotional burden reported, our study findings showed the absence of any relevant correlation between caregivers' burden and children's level of functioning. This suggests that these children are so severely affected that slight differences in functioning do not translate into differences into the amount of daily difficulties parents have to deal with. It can therefore be supposed that the severe disability of these children represents such a complex condition that slight functioning differences between children conditions do not differently impact the amount of daily difficulties parents have to deal with.

Limitations to this study include the sample size, which restricts the possibility to generalise the results to the whole population of children and adolescents with VS and MCS. One of the reasons for such a limited sample lies in the fact that this population of patients is not usually hosted in long-term rehabilitation centres and lives at home because parents prefer to take care of them at home, determining concrete difficulties in enrolment. It should be also considered that a sample composed of 35 children in VS and MCS is considered a large sample, based on available epidemiology of VS and MCS [4]. In addition, the intrinsic weakness of cross-sectional study design does not allow one to imply causal relationship between variables, and therefore longitudinal studies are planned.

Further possible limitation is referred to the heterogeneous professional backgrounds of researchers responsible for administering scales and questionnaires. A common training to standardise procedure with detailed instructions was provided to limit possible differences. Finally, a comment should also be made on the use of one sample *t*-test to assess differences between caregivers and normative sample with regard to STAI-Y scores. STAI-Y guidelines recommend to differentiate scores based on respondents' gender, and for this reason separate testing was performed dividing males and females. The fact that only five males were enrolled as caregivers is a serious shortcoming on the reliability of such a procedure.

To our knowledge, this is the only study reporting information on children in VS and MCS and their caregivers. Person-based rehabilitative programs focused on children's real needs and functioning are required to provide sensible and systemic interventions and enhance clinical and functional health status. Means for improving the network of psychological support and social assistance offered by community-based services and hospitals to the caregivers should be provided as this may contribute to decrease the burden of caregivers and improve caring abilities, and eventually children quality of life. Facilitating the use of emotion-focused strategies instead of problem-focussed strategies may represent an important first step to enhance caregiver's ability to cope with stressful situation and ameliorate parents' mental health.

## Figures and Tables

**Table 1:** Sociodemographic characteristics of children with DOCs.

Age, mean (SD)	8.9 (4.5)
Time from acute event, mean (SD)	4.2 (4.0)
Gender, *n* (%)	
Males	24 (68.6)
Females	11 (31.4)
Place of residence, *n* (%)	
Domicile	27 (77.1)
Post-acute rehabilitation centre	8 (22.9)
Long term care centre	0 (0)

**Table 2:** Clinical characteristics of children with DOCs.

Main diagnosis, *n* (%)	
SV	21 (60.0)
SMC	14 (40.0)
Comorbidities, *n* (%)	
Tetraplegia	18 (51.4)
Dysphagia	12 (34.3)
Epilepsy	10 (27.8)
Respiratory failure	1 (2.8)
Dwarfism	1 (2.8)
Severe mental retardation	1 (2.8)
Aetiology, no (%)	
Anoxia	16 (45.7)
Traumatic	11 (31.3)
Others	8 (23.0)
Rehabilitative interventions, *n* (%)	
Physiotherapy	32 (91.4)
Swallowing and language	10 (28.6)
Psychological and cognitive	5 (14.3)
Breathing	2 (5.7)
Music therapy	2 (5.7)
Disability rating scale, mean (SD)	22.0 (1.9)

**Table 3:** Socio-demographic characteristics of caregivers of children with DOCs.

Age, mean (SD)	38.7 (6.7)
Relationship to patient, *n* (%)	
Mother	30 (85.7)
Father	5 (14.3)
Education, *n* (%)	
Middle school or lower	2 (5.7)
High school	20 (57.1)
University degree or higher	13 (37.2)
Marital Status, *n* (%)	
Married	29 (82.9)
Single	2 (5.7)
Separated or widow	4 (11.4)
Employment status, *n* (%)	
Housewife	18 (51.4)
Blue collar	7 (20.0)
White collar	6 (17.1)
Other	4 (11.5)
Resignment from job, *n* (%)	
Yes, permanently	11 (31.4)
Yes, temporarily	12 (34.3)
No	12 (34.3)
Assistance, *n* (%)	
All day (24 h)	29 (82.9)
Daytime care	4 (11.4)
Night care	2 (5.7)
Psychological support, *n* (%)	
Yes	11 (31.4)
No	24 (68.6)
Social assistance, *n* (%)	
Yes	13 (37.1)
No	22 (62.9)
Perceived economic status, *n* (%)	
Poor	2 (5.7)
Sufficient	22 (62.9)
Medium	8 (22.9)
Well-off	3 (8.5)
Financial difficulties, *n* (%)	
Yes	21 (60.0)
No	14 (40.0)
Familiar support for caring, *n* (%)	
Yes	17 (48.6)
No	18 (51.4)

**Table 4:** Scores of psychological assessments of caregivers of children with DOCs.

BDI-II, *n* (%)	
Minimal	15 (42.8)
Mild	7 (20.0)
Moderate	3 (8.6)
Severe	10 (28.6)
SF-12, mean (SD)	
Physical component summary	51.6 (4.5)
Mental component summary	44.8 (8.4)
STAI-Y, mean (SD)	
State anxiety	45.6 (12.3)
Trait anxiety	47.2 (10.4)
COPE, mean (SD)	
Problem oriented strategies	51.4 (9.7)
Strategies of emotional expression	66.0 (10.7)
Potentially dysfunctional strategies	21.9 (4.4)
